# Clinical characteristics and prognostic value of MEX3A mRNA in liver cancer

**DOI:** 10.7717/peerj.8252

**Published:** 2020-01-21

**Authors:** Dingquan Yang, Yan Jiao, Yanqing Li, Xuedong Fang

**Affiliations:** 1 Department of Gastrointestinal Colorectal and Anal Surgery, China-Japan Union Hospital of Jilin University, Changchun, Jilin, China; 2 Department of Hepatobiliary and Pancreatic Surgery, The First Hospital of Jilin University, Changchun, Jilin, China; 3 Department of Pathophysiology, College of Basic Medical Sciences, Jilin University, Changchun, Jilin, China

**Keywords:** MEX3A, Liver cancer, Prognosis, RNA-binding proteins, The Cancer Genome Atlas

## Abstract

**Background:**

MEX3A is an RNA-binding proteins (RBPs) that promotes the proliferation, invasion, migration and viability of cancer cells. The aim of this study was to explore the clinicopathological characteristics and prognostic significance of MEX3A mRNA expression in liver cancer.

**Methods:**

RNA-Seq and clinical data were collected from The Cancer Genome Atlas (TCGA). Boxplots were used to represent discrete variables of MEX3A. Chi-square tests were used to analyze the correlation between clinical features and MEX3A expression. Receiver operating characteristic (ROC) curves were used to confirm diagnostic ability. Independent prognostic ability and values were assessed using Kaplan–Meier curves and Cox analysis.

**Results:**

We acquired MEX3A RNA-Seq from 50 normal liver tissues and 373 liver cancer patients along with clinical data. We found that MEX3A was up-regulated in liver cancer which increased according to histological grade (*p* < 0.001). MEX3A showed moderate diagnostic ability for liver cancer (AUC = 0.837). Kaplan–Meier curves and Cox analysis revealed that the high expression of MEX3A was significantly associated with poor survival (OS and RFS) (*p* < 0.001). Moreover, MEX3A was identified as an independent prognostic factor of liver cancer (*p* < 0.001).

**Conclusions:**

MEX3A expression shows promise as an independent predictor of liver cancer prognosis.

## Introduction

Liver cancer is a malignant cancer with poor prognosis that is responsible for more than 780,000 deaths annually, making it the second most common cause of cancer-related mortality worldwide (Bray et al., 2018). Although liver cancer can be alleviated or cured through hepatectomy, orthotopic liver transplantation, and/or ablative procedures (Hanouneh, Alkhouri & Singal, 2019). More than 65% of patients fail to be cured and frequent recurrence contributes to poor survival. Predicting the overall 1-year survival rates for liver cancer have remained a challenge (Galle et al., 2018; Hanouneh, Alkhouri & Singal, 2019). New biomarkers that can predict liver cancer recurrence are urgently required to improve prognosis.

Liver cancer is influenced by post-transcriptional mechanisms that dynamically regulate protein expression (Wong, Tsang & Ng, 2018; Goldstrohm, Hall & McKenney, 2018). Cis-regulatory RNA elements and trans-acting factors (Gerstberger, Hafner & Tuschl, 2014; Moore, 2005) including RNA-binding proteins (RBPs) play an essential role in gene expression in cancer cells (Masuda & Kuwano, 2019). Recently, a group of RBPS termed MEX-3 RNA binding family member (MEX3) was identified in the nematode Caenorhabditis elegans (Ciosk, DePalma & Priess, 2006) and revealed one of the few RBPs with carcinogenic or tumor suppressor activity (Kim, Hur & Jeong, 2009; Pereira et al., 2013a).

MEX3 proteins are evolutionarily conserved RNA-binding proteins that consist of four homologous genes (MEX3A–D) (Buchet-Poyau et al., 2007; Courchet et al., 2008; Pereira et al., 2013a). They contained highly conservative one carboxy-terminal RING finger module and two K homology domains, the former mediating E3 ubiquitin ligase activity, the latter providing RNA-binding capacity (Buchet-Poyau et al., 2007). Available evidence implicates the MEX3 family in epithelial homeostasis, embryonic development, metabolism, immune responses and cancer, but the specific mechanisms of these effects require elucidation (Pereira et al., 2013a). MEX3A is a member of the MEX3 family (also known as RKHD4 or RNF162) that is expressed in endometrium tissue and the ovaries. MEX3A is a novel component of GW-182 or Dcp-containing bodies in mammals that represent cellular sites of mRNA degradation and the sequestration of non-translated transcripts (Cougot, Babajko & Seraphin, 2004; Eystathioy et al., 2002; Sheth & Parker, 2003).

MEX3A mRNA was recently shown to be overexpressed in Wilms tumors (Krepischi et al., 2016), gastric cancer (Jiang et al., 2012), bladder cancer (Huang et al., 2017), and bladder urothelial carcinoma (Shi & Huang, 2017). MEX3A promotes cell proliferation in bladder (Huang et al., 2017) and gastric (Jiang et al., 2012) cancer and shows potential as a biomarker to predict carcinogenesis (Pereira et al., 2013a). In this study, we analyzed the expression of MEX3A in liver cancer and assessed its clinicopathological potential. We further investigated the potential of MEX3A as an independent predictor of liver cancer prognosis.

## Methods

### Clinical and RNA-Seq analysis

We downloaded the all RNA-Seq expression matrix from the Cancer Genome Atlas (TCGA) database and obtained MEX3A mRNA expression data from liver cancer vs. normal liver tissue using the matrix. We further obtained corresponding clinical and pathological information from TCGA database. The basic clinical data included age, gender, histological grade, stage, *T*∕*N*∕*M* classification and vital status. MEX3A mRNA expression were estimated as log2(x+1) values and transformed RSEM normalized counts.

### Statistical analyses

We retrospectively analyzed all data using R (version 3.5.1) (R Core Team, 2009). We used non-parametric rank sum tests to analyze MEX3A mRNA expression levels according to different variables and boxplots were visualized. Wilcoxon rank sum tests were used to compare the two subgroups, including disease, age, gender and vital status. Kruskal–Wallis tests were used for the comparison of three or more subgroups, including clinical stage, histologic grade and *T*∕*N*∕*M* classification. We used the pROC package to draw ROC curves for the evaluation of MEX3A diagnosis through the calculation of AUC values and the measurement of optimal cutoff point to divide samples into high and low MEX3A expression groups (Robin et al., 2011). Further, correlation between clinical features and MEX3A expression group were analyzed through chi-square tests with Fisher’s exact test.

To evaluate prognosis, Kaplan–Meier curves were used based on log-rank tests to compare differences in survival status, including overall survival (OS) and relapse-free survival (RFS) between the high and low MEX3A expression groups using the survival package in R (Therneau, 1994; Therneau & Grambsch, 2000). Univariate Cox analysis was used to select factors associated with prognosis, with calculations of hazard ratios (HRs) and 95% confidence intervals (95% CIs). Independent prognostic values of OS and RFS in the patients were determined through Multivariate Cox analysis. *P*-values < 0.05 were deemed statistically significant.

## Results

### Clinical characteristics and RNA-Seq analysis

A total of 423 tissue samples with MEX3A mRNA expression data, including 373 liver cancer and 50 normal liver tissues were obtained from the TCGA. All patients were diagnosed with primary liver cancer. Corresponding patient demographic and clinical characteristics such as age, gender, histologic grade, TNM stage, vital status, and radiation therapy were obtained. All patient data is shown in Table 1.

**Table 1:** MEX3A mRNA expression and clinical characteristics in liver cancer patients.

Characteristics	Number of pts(%)
Age	
<55	117(31.45)
>=55	255(68.55)
NA	1(0.00)
Gender	
Female	121(32.44)
Male	252(67.56)
Histological_type	
Fibrolamellar Carcinoma	3(0.8)
Hepatocellular Carcinoma	363(97.32)
Hepatocholangiocarcinoma (Mixed)	7(1.88)
Histologic_grade	
G1	55(14.75)
G2	178(47.72)
G3	123(32.98)
G4	12(3.22)
NA	5(1.34)
Stage	
I	172(46.11)
II	87(23.32)
III	85(22.79)
IV	5(1.34)
NA	24(6.43)
T_classification	
T1	182(48.79)
T2	95(25.47)
T3	80(21.45)
T4	13(3.49)
TX	1(0.27)
NA	2(0.54)
N_classification	
N0	253(67.83)
N1	4(1.07)
NX	115(30.83)
NA	1(0.27)
M_classification	
M0	267(71.58)
M1	4(1.07)
MX	102(27.35)
Radiation_therapy	
No	340(91.15)
Yes	8(2.14)
NA	25(6.7)
Residual_tumor	
R0	326(87.4)
R1	17(4.56)
R2	1(0.27)
RX	22(5.9)
NA	7(1.88)
Vital_status	
Deceased	130(34.85)
Living	243(65.15)
Relapse	
No	179(55.94)
Yes	141(44.06)
NA	53(14.2)
MEX3A	
High	117(31.37)
Low	256(68.63)

**Notes.**

NA, not available.

### MEX3A is highly expressed in liver cancer

**Figure 1:**
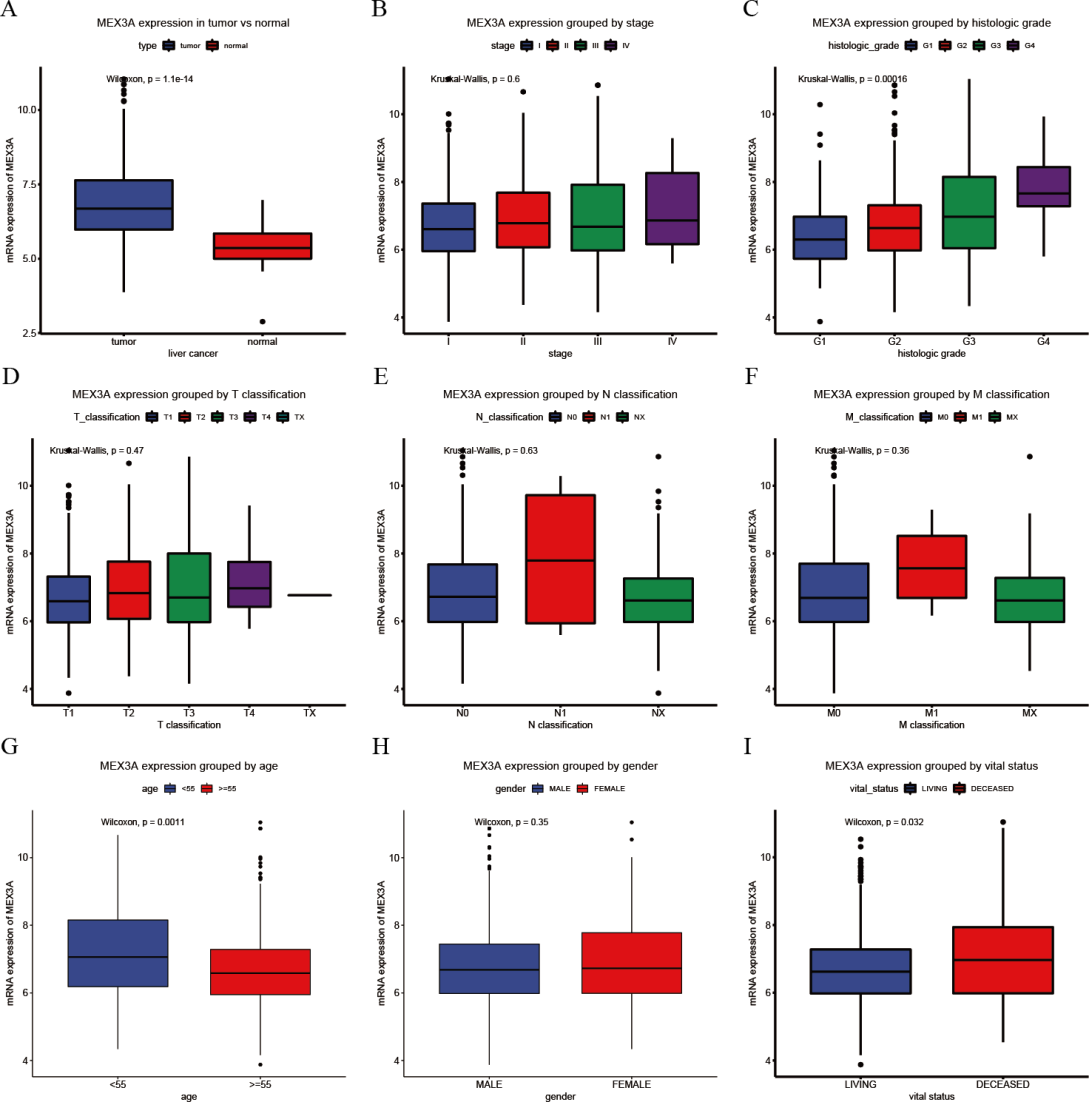
Assessment of the relationship between MEX3A mRNA expression and clinical characteristics (A–I). Comparison of MEX3A mRNA expression in 373 cases of liver cancer and 50 normal liver tissues (A). Comparison of MEX3A mRNA expression according to clinical parameters: clinical stage (I, II, III and IV) (B), histologic grade (G1, G2, G3 and G4) (C), T classification (T1, T2, T3 and T4) (D), N classification (N0, N1 and NX) (E), M classification (M0, M1 and MX) (F), age (<55 and ≥55) (G), gender (male and female) (H) and vital status (I).

In liver cancer, MEX3A mRNA expression level was significantly up-regulated compared to normal tissues (*p* = 1.1e−14; Fig. 1A) and increased with higher histological grades (*p* = 0.00016; Fig. 1C). MEX3A expression level was significantly associated with vital status (*p* = 0.032; Fig. 1I) and age (*p* = 0.0011; Fig. 1G).

### MEX3A as a liver cancer diagnostic.

**Figure 2:**
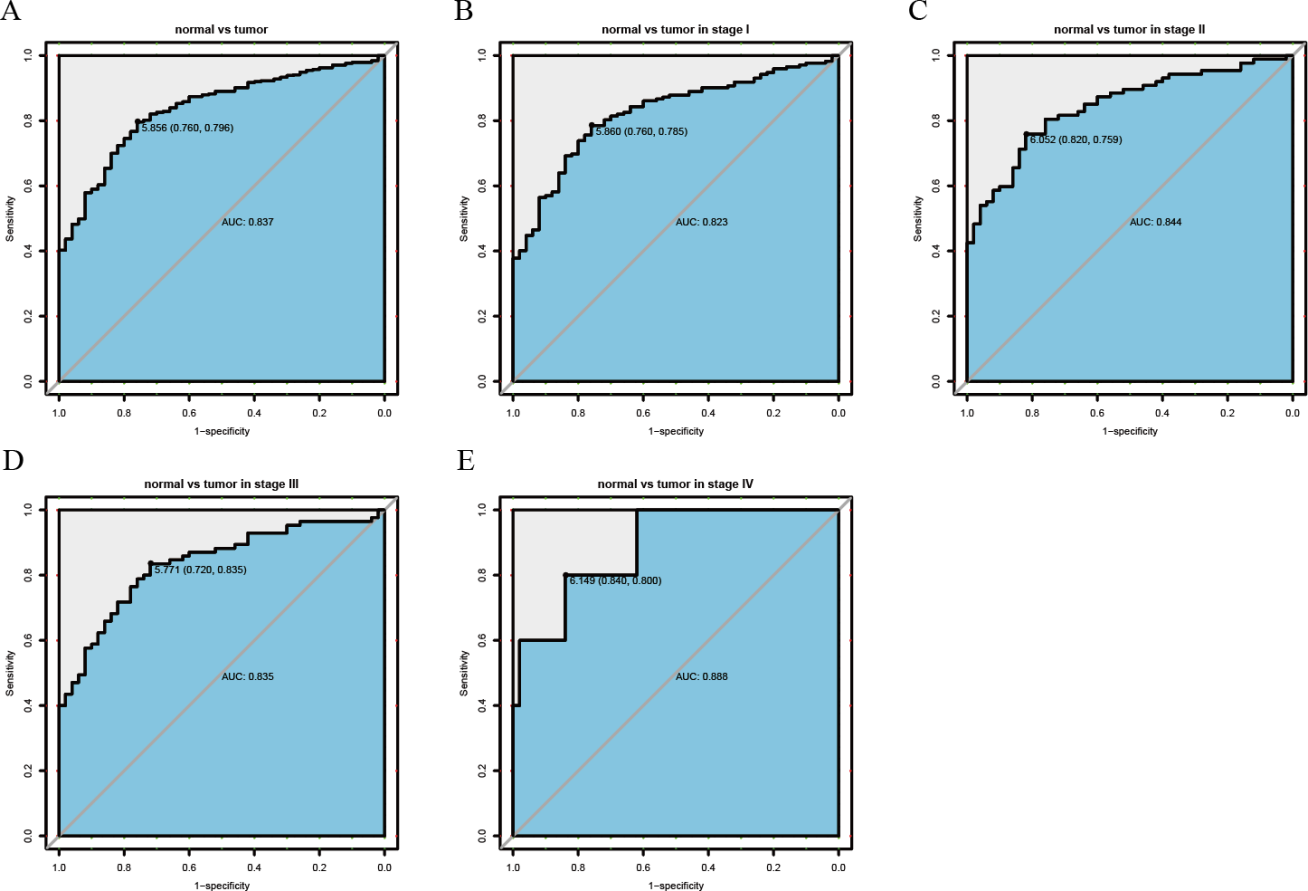
ROC analysis of the sensitivity and specificity of MEX3A to assess liver cancer. ROC curve for MEX3A expression in normal liver tissue and tumor tissue: AUC = 0.837 (A). I stage patients: AUC = 0.823 (B). II stage patients: AUC = 0.844 (C). III stage patients: AUC = 0.835 (D). IV stage patients: AUC = 0.888 (E). Abbreviations: AUC, area under the curve; ROC, receiver-operating characteristics.

ROC curve analysis showed that MEX3A had moderate diagnostic ability in patients with liver cancer (AUC=0.837; Fig. 2A). The diagnostic ability of MEX3A was comparable in all clinical stages (stage I: AUC = 0.823, stage II: AUC = 0.844, stage III: AUC = 0.835, stage IV: AUC = 0.888; Figs. 2B–2E).

### Relationship between MEX3A expression and clinical characteristics

Figure S1 showed that the cutoff point was 7.266 when samples were divided according to high and low MEX3A expression. Chi-square tests were used to analyze clinical variables between the two groups, in which high MEX3A expression was associated with cancer related mortality (*p* = 0.001; Table 2). High MEX3A expression was also associated with a deterioration in liver tumor histopathology (*p* < 0.001; Table 2).

**Table 2:** Association between MEX3A expression and clinical characteristics in liver cancer patients.

Clinical characteristics			MEX3A expression					
	Variable	No. of patients	High	%	Low	%	*χ*2	*p*-value
Age	<55	117	51	43.59	66	25.88	10.8573	**0.001**
	>=55	255	66	56.41	189	74.12		
Gender	Female	121	43	36.75	78	30.47	1.1741	0.279
	Male	252	74	63.25	178	69.53		
Histological type	Fibrolamellar Carcinoma	3	0	0	3	1.17	1.8006	0.547
	Hepatocellular Carcinoma	363	114	97.44	249	97.27		
	Hepatocholangiocarcinoma (Mixed)	7	3	2.56	4	1.56		
Histologic grade	G1	55	10	8.7	45	17.79	20.0434	**0.000**
	G2	178	48	41.74	130	51.38		
	G3	123	48	41.74	75	29.64		
	G4	12	9	7.83	3	1.19		
stage	I	172	48	43.64	124	51.88	2.1216	0.512
	II	87	30	27.27	57	23.85		
	III	85	30	27.27	55	23.01		
	IV	5	2	1.82	3	1.26		
T classification	T1	182	49	41.88	133	52.36	4.9613	0.253
	T2	95	35	29.91	60	23.62		
	T3	80	27	23.08	53	20.87		
	T4	13	6	5.13	7	2.76		
	TX	1	0	0	1	0.39		
N classification	N0	253	86	73.5	167	65.49	3.4688	0.135
	N1	4	2	1.71	2	0.78		
	NX	115	29	24.79	86	33.73		
M classification	M0	267	88	75.21	179	69.92	2.0953	0.304
	M1	4	2	1.71	2	0.78		
	MX	102	27	23.08	75	29.3		
radiation therapy	NO	340	106	98.15	234	97.5	0	1.000
	Yes	8	2	1.85	6	2.5		
Residual tumor	R0	326	97	84.35	229	91.24	5.1533	0.134
	R1	17	7	6.09	10	3.98		
	R2	1	0	0	1	0.4		
	RX	22	11	9.57	11	4.38		
Vital status	Deceased	130	55	47.01	75	29.3	10.3281	**0.001**
	Living	243	62	52.99	181	70.7		

**Notes.**

% represents the distribution of different clinical features in the single MEX3A expression group *P*-value in bold represent significant clinical significance (*p* < 0.05).

### MEX3A is an independent prognostic to evaluate the survival of liver cancer patients

**Figure 3:**
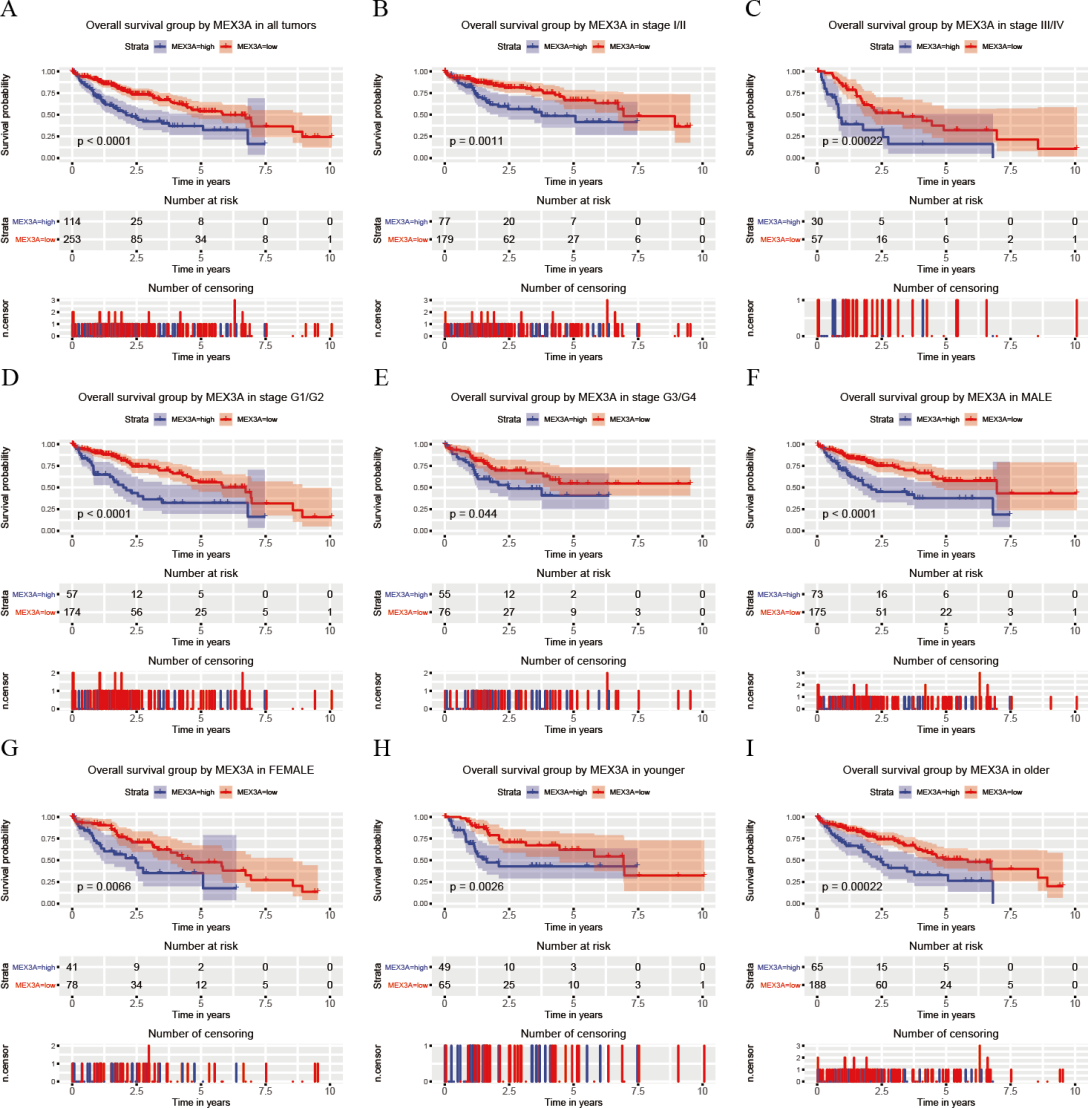
Analysis of OS between high and low expression groups of MEX3A according to the different clinical variables of liver cancer patients. Kaplan-Meier curves of OS in all patients with liver cancer (A). Subgroup analysis was performed in stage I/II (B), stage III/IV (C), histological grade G1/G2 (D), histological grade G3/G4 (E), males (F), females (G), young patients (H) and old patients (I).

Kaplan–Meier curves showed that patients with high MEX3A expression were more likely to have a poor OS (*p* < 0.0001; Fig. 3A). Further subgroup analysis showed that high MEX3A expression was associated with poor OS for all variables: stage I/II (*p* = 0.0011; Fig. 3B), stage III/IV (*p* = 0.00022; Fig. 3C), stage G1/G2 (*p* < 0.0001; Fig. 3D), stage G3/G4 (*p* = 0.044; Fig. 3E), male (*p* < 0.0001; Fig. 3F), female (*p* = 0.0066; Fig. 3G), younger (*p* = 0.0026; Fig. 3H), older (*p* = 0.00022; Fig. 3I). Through Univariate and Multivariate Cox analysis of OS, MEX3A (HR = 2.26, 95% CI [1.58–3.23], *p* < 0.0001) was identified as an independent risk factor for the prognosis of liver cancer along with T stage (*p* < 0.0001) and residual tumors (*p* = 0.026; Table 3).

**Table 3:** Relationship between clinical parameters, MEX3A mRNA expression and overall survival in liver cancer patients.

	Univariate analysis			Multivariate analysis		
Parameters	Hazard ratio	95%CI (lower∼upper)	*P* value	Hazard ratio	95%CI (lower–upper)	*P* value
Age	1.00	0.69–1.45	0.997			
Gender	0.80	0.56–1.14	0.220			
Histological type	0.99	0.27–3.66	0.986			
Histologic grade	1.04	0.84–1.3	0.698			
Stage	1.38	1.15–1.66	0.001	0.86	0.69–1.07	0.163
T classification	1.66	1.39–1.99	0.000	1.88	1.48–2.38	**0.000**
N classification	0.73	0.51–1.05	0.086			
M classification	0.72	0.49–1.04	0.077			
Radiation therapy	0.51	0.26–1.03	0.060			
Residual tumor	1.42	1.13–1.8	0.003	1.33	1.03–1.71	**0.026**
MEX3A	2.29	1.61–3.26	0.000	2.26	1.58–3.23	**0.000**

**Notes.**

*P*-value in bold represent significant clinical significance (*p* < 0.05)

**Figure 4:**
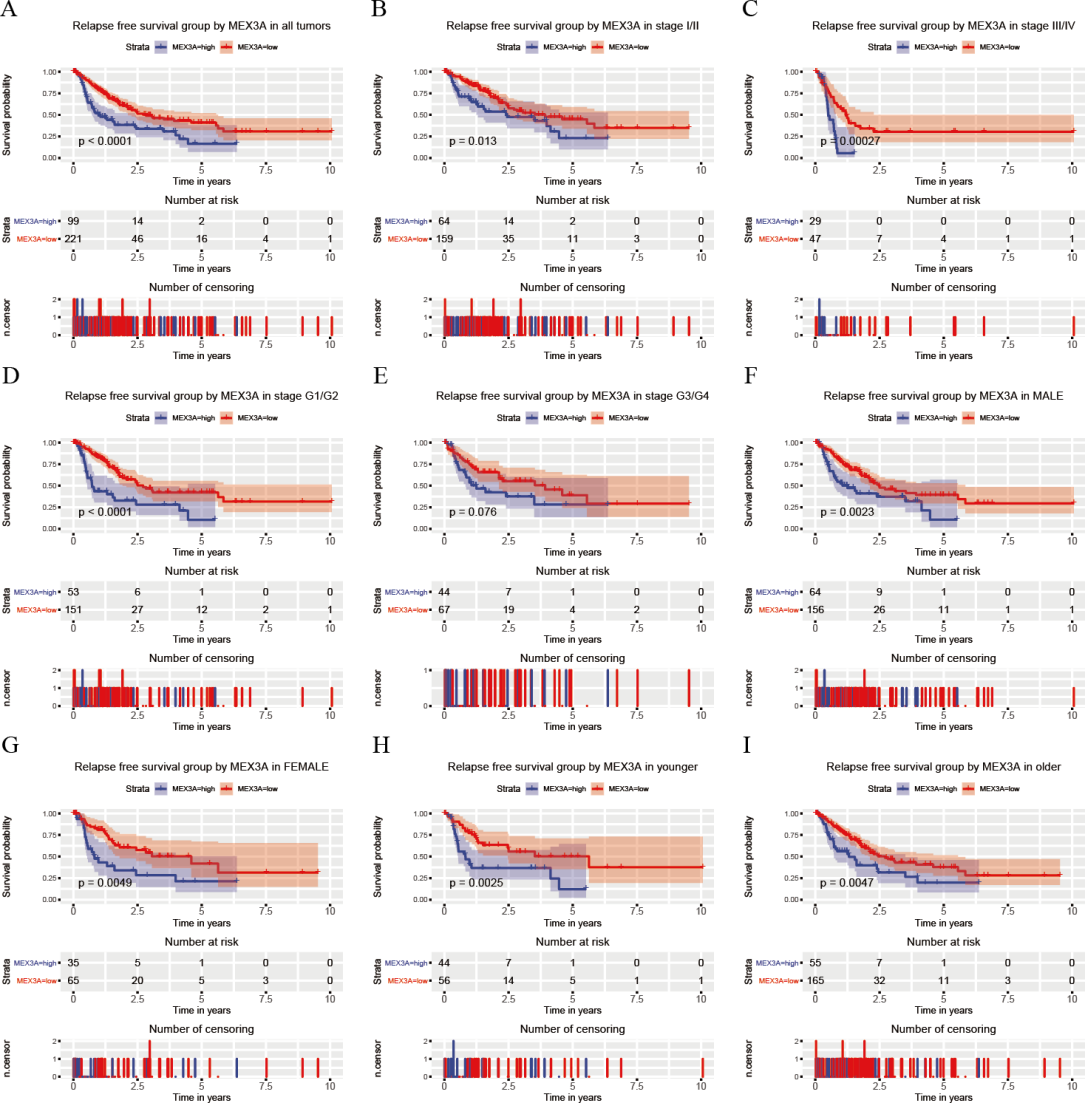
Analysis of RFS in high and low MEX3A expression groups according to the clinical variables of liver cancer patients. Kaplan-Meier curves of RFS in all patients with liver cancer (A). Subgroup analysis was performed in stage I/II (B), stage III/IV (C), histological grade G1/G2 (D), histological grade G3/G4 (E), males (F), females (G), young patients (H) and old patients (I).

Based on the OS, we further explored the connection between RFS and MEX3A expression, and found that high MEX3A expression was associated with poor RFS (*p* < 0.0001; Fig. 4A). RFS was related to the expression of MEX3A for some variables, including stage I/II (*p* = 0.013; Fig. 4B), stage III/IV (*p* = 0.00027; Fig. 4C), stage G1/G2 (*p* < 0.0001; Fig. 4D), male (*p* = 0.0023; Fig. 4F), female (*p* = 0.0049; Fig. 4G), younger (*p* = 0.0025; Fig. 4H) and older (*p* = 0.0047; Fig. 4I). Univariate and Multivariate Cox analysis suggested that MEX3A (HR = 2.19, 95% CI [1.54–3.12], *p* < 0.0001) was an independent risk factor to evaluate the RFS for liver cancer along with T stage (*p* < 0.0001) and residual tumor status (*p* = 0.024; Table 4).

## Discussion

Liver cancer is one of the deadliest tumors worldwide. The main risk factors for liver cancer include hepatitis B or C virus infections, the intake of aflatoxin, alcohol abuse and non-alcoholic fatty liver disease (NAFLD) (Zhang, Yang & Ericsson, 2019). Due to the lack of effective diagnostic and prognostic evaluation methods, the mortality rates of liver cancer patients have gradually increased. New molecular markers that can guide prognosis and improve the survival rates of liver cancer patients are urgently required. Our team has devoted to exploring diagnostic and prognostic biomarkers in various cancers (Hou et al., 2019; Jiao et al., 2018; Jiao et al., 2019a; Jiao et al., 2019b; Jiao et al., 2019c; Jiao et al., 2019d; Li et al., 2019; Sun et al., 2019). In this study, MEX3A mRNA was identified as overexpressed in liver cancer tissue and could effectively evaluate the prognosis of liver cancer patients as an independent predictor. A strong correlation between high MEX3A expression and liver malignancy was also observed.

MEX3A is known to be upregulated in Wilms renal cancer (Krepischi et al., 2016), gastric cancer (Jiang et al., 2012), bladder cancer (Huang et al., 2017) and bladder urothelial cancer (Shi & Huang, 2017). These results are consistent with our finding that MEX3A mRNA is overexpressed in liver cancer (*p* = 1.1e^−14^). Area under the ROC curves was 0.837, which provided evidence that MEX3A was a potential biomarker for liver cancer diagnosis. Interestingly, the expression of MEX3A was higher with increased histological grade (*p* < 0.0001), suggesting that MEX3A is related to tumor progression.

The molecular mechanism underlying the oncogenic effects of MEX3A remain poorly understood. Huang et al. (2017) found that MEX3A silencing significantly inhibits the proliferation of bladder cancer cells and promotes apoptosis. Jiang et al. similarly reported that MEX3A silencing delays the cell cycle progression of gastric cancer cells. MEX3A silencing significantly inhibited cell migration and anchorage-independent growth (Jiang et al., 2012). In colorectal cells, MEX3A is a stemness-related gene (Barriga et al., 2017; Chatterji & Rustgi, 2018; Fernandez-Barral et al., 2019) that acts as a repressive factor through controlling the expression of CDX2. CDX2 inhibits colorectal tumor cells growth, invasion, progression and migration and plays an essential regulatory role in intestinal homeostasis (Bonhomme et al., 2003; Brabletz et al., 2004; Gross et al., 2008; Pereira et al., 2013b). If MEX3A is overexpressed in colonic cell lines, cell polarity and differentiation become impaired leading to carcinogenesis (Pereira et al., 2013b). Combined with our findings, the role of MEX3A in cancer progression explains its clinical links to poor histological grade and poor patient prognosis in liver cancer. It is therefore necessary to explore the relationship between MEX3A and survival.

The MEX3 family shows promise as a biomarker for both cancer progression and prognosis (Pereira et al., 2013a). However, in studies by Huang et al. (Shi & Huang, 2017), no prognostic significance for cancer bladder urothelial carcinoma samples following MEX3A overexpression were observed. These results contrasted our findings and may highlight differential roles of MEX3A in cancer progression according to cancer-type. Of note, upon assessment of each sub-variable group, Kaplan–Meier curves revealed that the liver cancer patients with high MEX3A expression had a poor OS. RFS were evaluated according to MEX3A expression and showed a similar relationship with OS, excluding G3/G4 group. This highlights the unique superiority of MEX3A expression for the assessment of liver cancer survival. In particular, as shown in Tables 3 and 4, MEX3A may represent a prognostic marker for liver cancer survival (OS and RFS) under the strong confounding effects of clinicopathological features, providing useful references for clinicians to aid the development of individualized patient’s treatments.

In summary, this is the first study to report the association between MEX3A mRNA and the clinical characteristics and survival of liver cancer patients. MEX3A has great potential to predict the prognosis of liver cancer patients. Future studies should explore the mechanisms by which MEX3A promotes liver cancer *in vivo* and *in vitro*. Further clinicopathological information and corresponding clinical tissue samples should be obtained to further validate these findings and to establish MEX3A as a novel prognostic for patients with liver cancer.

**Table 4:** Relationship between clinical parameters, MEX3A mRNA expression and relapse-free survival in liver cancer patients.

	Univariate analysis			Multivariate analysis		
Parameters	Hazard ratio	95%CI (lower∼upper)	*P* value	Hazard Ratio	95%CI (lower–upper)	*P* value
Age	0.90	0.63–1.28	0.550			
Gender	0.99	0.7–1.41	0.966			
Histological type	2.02	0.66–6.24	0.220			
Histologic grade	0.98	0.8–1.21	0.883			
Stage	1.66	1.38–1.99	0.000	1.13	0.87–1.46	0.358
T classification	1.78	1.49–2.12	0.000	1.69	1.29–2.21	**0.000**
N classification	0.97	0.67–1.4	0.874			
M classification	1.17	0.79–1.74	0.432			
Radiation therapy	0.74	0.26–2.16	0.584			
Residual tumor	1.28	1.01–1.61	0.042	1.32	1.04–1.67	**0.024**
MEX3A	2.05	1.46–2.9	0.000	2.19	1.54–3.12	**0.000**

**Notes.**

*P*-value in bold represent significant clinical significance (*p* < 0.05).

## Conclusions

This is the first study to investigate the expression of MEX3A mRNA in liver cancer, revealing its association with specific clinical features. Moreover, our results indicate that MEX3A plays a significant role in the prognosis of liver cancer and can be used as an independent factor to predict liver cancer progression.

##  Supplemental Information

10.7717/peerj.8252/supp-1 Figure S1 Optimal cutoff points for dividing patients into high and low MEX3A expression groups identified through ROC curves

## Additional Information and Declarations

### Data Availability

The following information was supplied regarding data availability: The raw data is available from the TCGA: https://xenabrowser.net/datapages/?cohort=TCGA%20Liver%20Cancer%20(LIHC)&removeHub=https%3A%2F%2Fxena.treehouse.gi.ucsc.edu%3A443%22.
