# Supplementary figures and images for: Clinical characteristics and prognostic value of MEX3A mRNA in liver cancer
Source: PeerJ. 2020 Jan 21;8:e8252. doi: 10.7717/peerj.8252 (PMC6979405; doi:10.7717/peerj.8252)

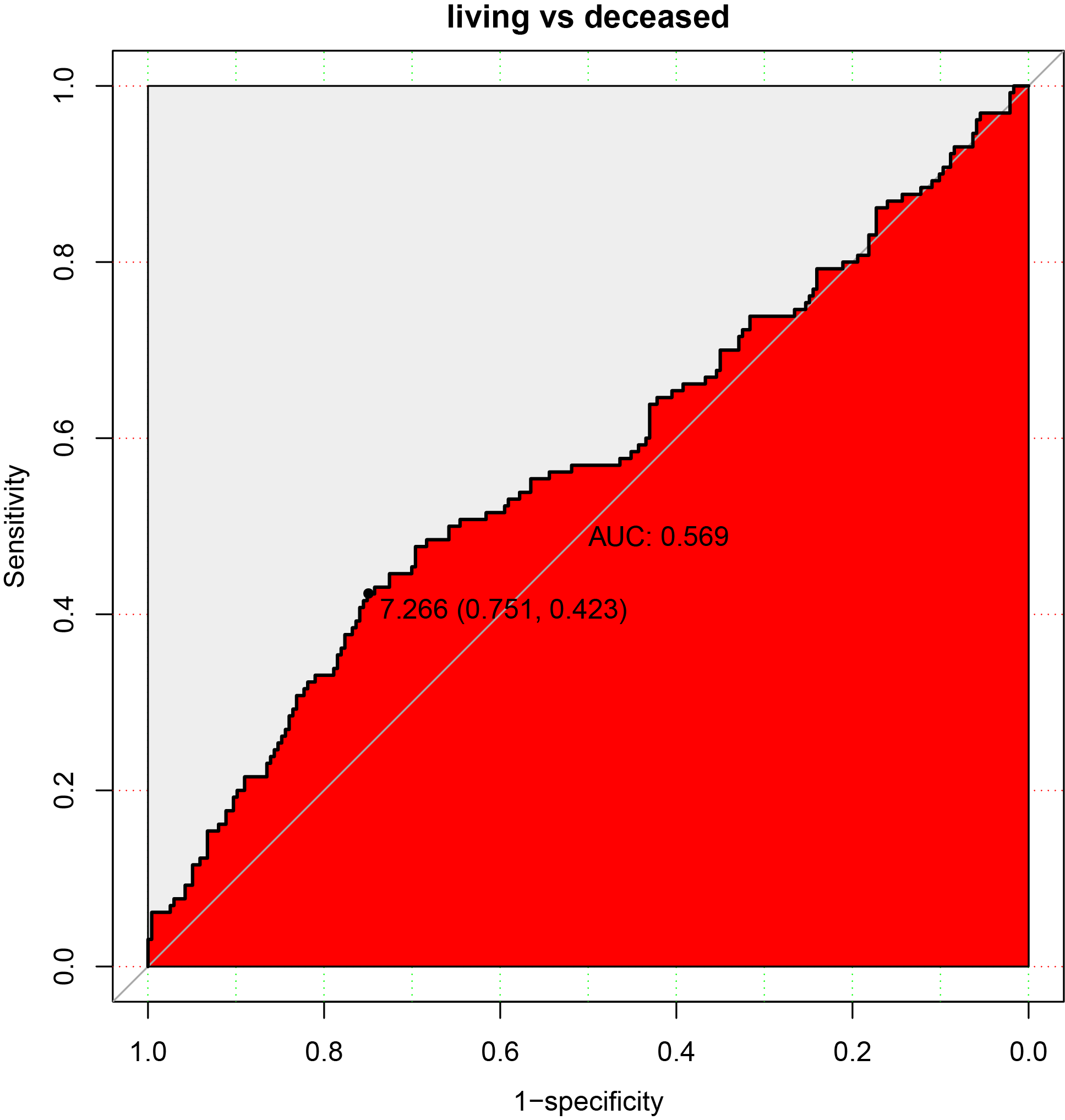

Supplement: Figure S1 [file peerj-08-8252-s001.png]
